# Adenovirus Type 7 Pneumonia in Children Who Died from Measles-Associated Pneumonia, Hanoi, Vietnam, 2014

**DOI:** 10.3201/eid2204.151595

**Published:** 2016-04

**Authors:** Le Thanh Hai, Hoang Ngoc Thach, Ta Anh Tuan, Dao Huu Nam, Tran Minh Dien, Yuko Sato, Toshio Kumasaka, Tadaki Suzuki, Nozomu Hanaoka, Tsuguto Fujimoto, Harutaka Katano, Hideki Hasegawa, Shoji Kawachi, Noriko Nakajima

**Affiliations:** The National Hospital of Pediatrics, Hanoi, Vietnam (L.T. Hai, H.N. Thach, T.A. Tuan, D.H. Nam, T.M. Dien);; National Institute of Infectious Diseases, Tokyo, Japan (Y. Sato, T. Suzuki, N. Hanaoka, T. Fujimoto, H. Katano, H. Hasegawa, N. Nakajima);; Japanese Red Cross Medical Center, Tokyo (T. Kumasaka);; Tomakomai City Hospital, Tomakomai, Japan (S. Kawachi)

**Keywords:** measles, adenovirus infection, secondary infection, pneumonia, viruses, children, Vietnam

## Abstract

During a 2014 measles outbreak in Vietnam, postmortem pathologic examination of hospitalized children who died showed that adenovirus type 7 pneumonia was a contributory cause of death in children with measles-associated immune suppression. Adenovirus type 7 pneumonia should be recognized as a major cause of secondary infection after measles.

Measles remains a fatal infectious disease, particularly among unvaccinated children and malnourished and immunocompromised patients. Measles virus causes systemic infection, and measles-related complications have been observed in every organ system (*1*). Pneumonia is one of the most common fatal complications and is caused by measles virus alone or by secondary viral and bacterial infections (*2*). Human adenovirus (AdV), human herpes virus, *Klebsiella* spp., *Pseudomonas* spp., and *Staphylococcus aureus* are commonly identified in the autopsied lung tissues of patients who died from measles-associated pneumonia (*3*,*4*).

In 2014, a measles outbreak occurred among mostly unvaccinated children in Vietnam; ≈15,000 confirmed cases and 146 deaths were reported (*5*). During January–October, a total of 2,462 patients with laboratory-confirmed measles infection were admitted to the National Hospital of Pediatrics (NHP) in Hanoi, Vietnam; 124 patients died (case-fatality rate 5%). Measles was diagnosed based on the presence of measles-specific IgM or the detection of measles RNA by reverse transcription PCR (RT-PCR). All 124 patients died from severe pneumonia. One patient’s illness was complicated with measles encephalitis; however, the main cause of death was severe pneumonia. To elucidate the underlying causes of death in children with measles who were admitted to NHP during the outbreak, we examined formalin-fixed and paraffin-embedded (FFPE) postmortem lung tissue samples by pathologic and molecular methods.

## The Study

During February–June 2014, postmortem lung biopsies were performed on 16 children (9 boys and 7 girls) who died in the NHP pediatric intensive care unit (PICU) from measles-associated pneumonia, defined as pneumonia within 30 days of rash onset. Although we attempted to obtain written, informed consent for the postmortem biopsies from the parents or legal guardians of all 124 patients, the parents/guardians of only 16 children consented. The study was approved by the Biomedical Research Ethics Committee of NHP (approval no. 14-012) and the Research and Ethics Committee of the National Institute of Infectious Diseases, Japan (approval no. 528).

Clinical and laboratory information on the patients was reviewed retrospectively (Table 1). Median age of patients was 8 months (range 4–16 months); all 16 patients were previously healthy and unvaccinated against measles. Median duration from disease onset to PICU admission was 9.5 days and from disease onset to death was 17.5 days. Co-infections with various bacteria, *Candida *spp., AdV, and cytomegalovirus (CMV) were detected in some patients (Table 1). Histopathologic studies (Table 2) revealed diffuse alveolar damage (Figure, panel A), necrotizing pneumonia (Figure, panel B), and organizing pneumonia (data not shown) in 12, 11, and 3 patients, respectively. Bacterial pneumonia was found in 1 patient (patient 16) (Tables 1, 2). Syncytial giant cells with intracytoplasmic and intranuclear eosinophilic inclusions, which are typical findings in measles giant-cell pneumonia, were found only in patient 2 (Figure, panel C). Intranuclear eosinophilic inclusions with halo or basophilic amorphous inclusion bodies with smudgy outlines, suggesting AdV infection, were found in 10 patients (patients 3–12) (Figure, panel D, inset).

**Table 1:** Clinical and laboratory characteristics of 16 children who died from measles-associated pneumonia in a pediatric intensive care unit, National Hospital of Pediatrics, Hanoi, Vietnam, February–June 2014*

Pt. no.	Age, mo/sex	Days from onset to		CD4/μL (CD4 %)	MEAS IgM†	Laboratory test results (type of test)			
		PICU admission	Death			NPA/TLA (culture)	Blood (rPCR)	AdV TLA (PCR)	CMV/mL blood (rPCR)
1	6/M	5	6	530 (28)	+	–	–	ND	ND
2	8/M	4	6	538 (19)	+	–	–	–	1.90 × 10^5^
3	14/F	9	12	754 (15)	+	–	–	ND	ND
4	12/M	6	14	714 (31)	+	–	–	+	ND
5	8/F	15	17	341 (37)	+	–	ND	+	–
6	16/F	16	18	120 (34)	–	–	–	+	–
7	10/M	22	27	793 (47)	+	*Acinetobacter *spp.	*Acinetobacter *spp.	+	6.72 × 10^4^
8	8/M	28	30	1,084 (26)	+	–	–	ND	ND
9	15/M	10	14	2,578 (52)	+	–	*Candida tropicalis,* *C. parapsilosis*	+	1.10 × 10^4^
10	4/M	10	15	947 (46)	+	–	*Klebsiella pneumoniae, K. oxytoca, Enterobacter cloacae, E. aerogenes*	+	–
11	9/F	16	20	372 (29)	+	–	*C. albicans*	+	1.10 × 10^4^
12	7/F	11	27	472 (21)	–	–	*Pseudomonas aeruginosa,* *Enterococcus faecalis, Stenotrophomonas maltophilia*	–	–
13	5/F	5	21	202 (22)	+	–	–	–	2.03 × 10^4^
14	4/M	5	24	326 (12)	+	–	–	ND	4.79 × 10^5^
15	7/M	8	25	1,732 (49)	+	*K. pneumoniae*	*Staphylococcus haemolyticus*	+	3.20 × 10^3^
16	15/F	5	10	ND	+	*Staphylococcus aureus*	–	ND	ND


*+, positive; –, negative; AdV, adenovirus; CMV, cytomegalovirus; MEAS, measles virus, ND, not done; NPA, nasopharyngeal aspirate; Pt, patient; PICU, pediatric intensive care unit; rPCR, real-time PCR; TLA, trachea lavage aspirate. †Specimens from patients 6 and 12 were positive for measles virus RNA.

**Table 2:** Histologic findings and detection of virus genomes and antigens in postmortem lung tissues of 16 children who died from measles-associated pneumonia in a pediatric intensive care unit, National Hospital of Pediatrics, Hanoi, Vietnam, February–June 2014*

Analysis	Patient no.															
	1	2	3	4	5	6	7	8	9	10	11	12	13	14	15	16
Histology																
Diffuse alveolar damage	No	Yes	Yes	Yes	No	Yes	Yes	Yes	No	Yes	Yes	Yes	Yes	Yes	Yes	No
Necrotizing pneumonia	No	No	Yes	Yes	Yes	Yes	Yes	Yes	Yes	Yes	Yes	Yes	No	No	No	Yes
Interstitial pneumonia	Yes	No	No	No	No	No	No	No	No	No	No	No	No	No	No	No
Organizing pneumonia	No	No	No	No	No	No	No	No	No	No	No	No	Yes	Yes	Yes	No
Bacterial pneumonia	No	No	No	No	No	No	No	No	No	No	No	No	No	No	No	Yes
Viral Inclusion body	Yes	Yes	Yes	Yes	Yes	Yes	Yes	Yes	Yes	Yes	Yes	Yes	No	No	No	No
Virus genome in FFPE lung†																
Measles virus RNA	3+	NA	UD	UD	UD	UD	UD	UD	NA	NA	NA	NA	UD	UD	NA	UD
AdV7 DNA	UD	NA	5+	7+	6+	3+	5+	7+	NA	NA	NA	NA	UD	UD	NA	UD
Cytomegalovirus DNA	UD	NA	UD	UD	UD	UD	2+	UD	NA	NA	NA	NA	3+	4+	NA	UD
Immunohistochemistry																
Measles virus	+	+	–	–	–	–	–	–	–	–	–	–	–	–	–	–
AdV	–	–	+	+	+	+	+	+	+	+	+	+	–	–	–	–
Cytomegalovirus	–	–	–	–	–	–	–	–	–	–	–	–	+	–	–	–


*+, positive; –, negative; AdV; adenovirus; AdV7, adenovirus type 7; FFPE, formalin-fixed and paraffin-embedded; NA, not available because the specimen sizes were too small for analysis; RT-PCR, reverse transcription PCR; UD, under detection limit. †Virus genome copy numbers were quantified by using real-time RT-PCR or real-time PCR: 2+, 10^2^–10^3^ copies/μL; 3+, 10^3^–10^4^ copies/μL; 4+, 10^4^–10^5^ copies/μL; 5+, 10^5^–10^6^ copies/μL; 6+, 10^6^–10^7^ copies/μL; 7+, 10^7^–10^8^ copies/μL.

**Figure:**
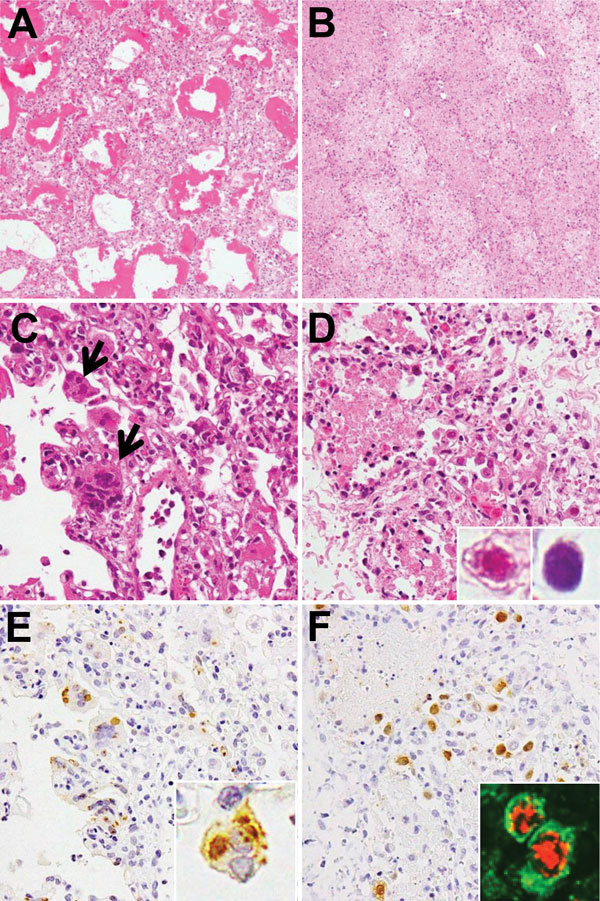
Histologic findings from postmortem lung tissues of children who died from measles-associated pneumonia in a pediatric intensive care unit, National Hospital of Pediatrics, Hanoi, Vietnam, January–October 2014. A) Diffuse alveolar damage with hyaline membrane formation (hematoxylin and eosin [H&E] stain, original magnification ×100). B) Necrotizing pneumonia with coagulation necrosis (H&E stain, original magnification ×100). C) Measles giant cell pneumonia. Arrows indicate syncytial cells with intracytoplasmic and intranuclear eosinophilic inclusions that were observed in the thickened alveolar walls (H&E stain, original magnification ×400). D) Adenovirus (AdV) pneumonia with necrotic epithelial cells and intranuclear inclusion bodies. Inset shows eosinophilic inclusion with halo and basophilic inclusion without halo (H&E stain, original magnification ×400). E) Measles nucleoprotein (brown) detected by immunohistochemistical analysis. Inset shows inclusions in syncytial cells with measles nucleoprotein (original magnification ×400). F) AdV antigen (brown) detected by immunohistochemistry (H&E stain, original magnification ×400). Inset: AdV antigens (red) were detected in the epithelial membrane antigen (green)–positive pneumocytes (double immunofluorescence stain, original magnification ×400).

We examined the virus genomes in the FFPE lung tissues by using molecular methods (*6*). First, RNA and DNA were extracted from the FFPE lung tissues of 10 patients. A multivirus real-time PCR (rPCR) system, which was used to screen for 163 types of virus genomes, detected measles virus RNA, AdV-subgroup B DNA, and CMV DNA. The copy numbers for each virus genome, along with internal reference genes, were quantified by using real-time RT-PCR (rRT-PCR) or rPCR (Table 2). To determine the serotype of the AdV-subgroup B, which was detected in 6 patients (patients 3–8), we sequenced the hypervariable regions of the hexon gene (784 nt) (*7*). Specimens from 5 of these 6 patients exhibited sequences that were consistent with AdV type 7 (AdV7; GenBank accession no. AC_000018), and the specimen from 1 patient exhibited a sequence with only 1 silent mutation.

To examine the distribution of virus antigens, we immunostained the lung sections with antibodies against each of the selected virus antigens. Measles virus nucleoprotein antigens were detected in the epithelial cells of patients 1 and 2 (Figure, panel E). AdV antigens were detected in all lung sections from 10 patients. Lung sections with higher copy numbers of AdV7 DNA had greater numbers of AdV antigen–positive cells (Figure, panel F). The infected cells appeared to be alveolar epithelial cells when observed after double immunofluorescence stain with epithelial membrane antigen (Figure, panel F, inset). Neither CMV inclusion bodies nor CMV antigens were observed in the CMV DNA–positive lung sections. Pathologic evidence of CMV disease was not obtained.

## Conclusions

The estimated national mortality rate for the 2014 measles outbreak in Vietnam was 1% (*5*). The case-fatality rate at NPH (5%) was disproportionately higher. There are at least 3 possible reasons for the high case-fatality rate in our hospital. First, we received referred patients with severe and complicated measles from provincial hospitals in northern Vietnam. Second, the number of patients exceeded the capacity of our hospital and made it impossible to properly isolate patients with measles. Third, the patients with measles at NPH easily acquired secondary bacterial or viral infections because of the 2 aforementioned conditions.

Although autopsies are not commonly performed in Vietnam, our use of postmortem biopsies was critical for understanding the underlying contributors to measles-associated deaths, at least among the deceased children who were evaluated. Our findings showed that 10 of the 16 patients demonstrated characteristic findings of severe AdV7 pneumonia: distinctive necrotizing lesions, numerous inclusion bodies, and AdV antigens.

AdV infection commonly occurs in children. It is generally mild or subclinical and thus is not problematic. However, AdV7 can cause serious and fatal disease, particularly in closed populations such as hospitalized children and military recruits (*8*,*9*). In addition, immunocompromised children may have a much higher risk for infection with AdV disease compared with their healthy counterparts because they lack an effective cell-mediated immune response (*10*). Measles causes immune suppression that can last from several weeks to several months (*11*). In approximately half the children in this study, the percentage of CD4-positive T cells decreased to <30% of the total number of lymphocytes at PICU admission, which suggests that measles-associated immune suppression may have resulted in increased susceptibility to AdV7 infection (*12*) (Table 1). The AdV7 hypervariable region sequences that were detected in our study were the same as those found in a similar study conducted in Singapore (*13*). AdV7 should be recognized as a potentially coinfectious pathogen in patients with measles. To ensure a rapid diagnosis, AdV virus load determination and virus typing should be performed for patients who are AdV DNA–positive by PCR.

The results of our study are subject to at least 3 limitations. First, our sample size was small and included only 16 of 124 measles-associated deaths that occurred at NPH in 2014; thus, we cannot be certain that our results are representative of all of the measles-associated deaths that occurred at the hospital. Second, because a limited portion of the lung was examined, other pathologic findings may have been missed. Third, we do not have epidemiologic data to rule out a concurrent outbreak of AdV7 infection in NHP or in Hanoi.

The extent to which the complication of AdV7 infection was responsible for the increase in the case-fatality rate is unknown. However, the postmortem pathologic examination of patients in our study showed that AdV7 coinfection was one of the co-morbid causes of death.
